# The Analytical Performance and Inter-laboratory Standardization of Next-Generation Sequencing (NGS) Panels for Genetic Risk Stratification in Acute Myeloid Leukemia: A Systematic Review and Meta-Analysis

**DOI:** 10.7759/cureus.103240

**Published:** 2026-02-08

**Authors:** Ayman Alqurain, Leen F Mashat, Najwa M Alshammari, Hashim A AL-Sumaily, Alqasem M Alamer, Reem S Aldhmadi, Muzun D Albalawi, Nada S Alghamdi, Ali S Metwaly

**Affiliations:** 1 Clinical Pharmacology and Therapeutics (Geriatric and Pain Management), Northern Border University, Arar, SAU; 2 Laboratory Science, Sheffaa Charity for Chronic Disease, Makkah, SAU; 3 Clinical Laboratories, College of Applied Medical Sciences, Hail University, Hail, SAU; 4 Medical Laboratory Technology, Dr. Sulaiman Al-Habib Hospital, Riyadh, SAU; 5 Laboratory, Mouwasat Hospital, Dammam, SAU; 6 Laboratory, New Hail Labs, Hail, SAU; 7 Medical Laboratory, Care Medical Hospital, Riyadh, SAU; 8 Microbiology Laboratory, National Guard Health Affair, Jeddah, SAU; 9 Medicinal Chemistry and Drug Discovery, Faculty of Pharmacy, Alexandria University, Alexandria, EGY

**Keywords:** acute myeloid leukemia, analytical validation, flt3-itd, genetic risk stratification, measurable residual disease, meta-analysis, next-generation sequencing

## Abstract

Genetic risk stratification is fundamental to the clinical management of acute myeloid leukemia (AML). While targeted next-generation sequencing (NGS) panels have replaced traditional single-gene assays, significant inter-laboratory variability in assay design and bioinformatic pipelines persists. This systematic review and meta-analysis evaluates the analytical performance and standardization of NGS panels used for AML risk stratification. Major databases were searched for studies reporting the analytical validation or inter-laboratory harmonization of NGS panels for AML compared to orthogonal reference standards (PCR/capillary electrophoresis). The primary outcomes were analytical concordance (overall percent agreement) and diagnostic accuracy. Risk of bias was assessed using Quality Assessment of Diagnostic Accuracy Studies-2 (QUADAS-2). A random-effects meta-analysis was conducted using the Freeman-Tukey double arcsine transformation to pool concordance rates. Heterogeneity was explored via meta-regression of assay limit of detection (LOD). The certainty of evidence was evaluated using Grading of Recommendations, Assessment, Development, and Evaluation (GRADE). Seven studies comprising 775 patient samples met the inclusion criteria. The pooled analytical concordance between NGS and reference standards was 96.4% (95% CI: 86.9%-100.0%). Diagnostic accuracy was excellent, with a hierarchical summary receiver operating characteristic (HSROC) area under the curve of 0.98. However, significant heterogeneity was observed (I^2^ = 91.9%, p < 0.0001). Meta-regression indicated that assay sensitivity (LOD) was a significant moderator; highly sensitive NGS assays (LOD ≤ 10^-4^) frequently detected low-level variants missed by standard PCR, leading to apparent discordance. Inter-rater reliability was strong (Global κ = 0.876). Targeted NGS panels demonstrate superior analytical sensitivity and high concordance with traditional methods, supporting their use as the new gold standard for AML risk stratification and measurable residual disease (MRD) monitoring. However, substantial inter-laboratory heterogeneity highlights the critical need for standardized reporting thresholds and harmonized bioinformatic pipelines to ensure consistent clinical interpretation.

## Introduction and background

Acute myeloid leukemia (AML) is a clonal hematopoietic malignancy characterized by biological heterogeneity and variable clinical outcomes [1,2]. AML pathogenesis is driven by the stepwise acquisition of somatic mutations, chromosomal aneuploidies, and fusion genes that disrupt normal differentiation and promote proliferation [3]. The clinical management of AML has shifted from a one-size-fits-all cytotoxic approach to personalized strategies contingent on precise molecular characterization [4]. The identification of recurrent driver mutations in genes such as NPM1, FLT3, CEBPA, TP53, RUNX1, and ASXL1 has become fundamental to prognostication, as codified by the European LeukemiaNet (ELN) recommendations [5,6].

The 2017 and 2022 ELN risk stratification guidelines emphasize the integration of cytogenetic and molecular data to categorize patients into favourable, intermediate, and adverse risk groups [5,6] as the prognostic impact of NPM1 mutations is modulated by the presence and allelic ratio of FLT3-internal tandem duplications (FLT3-ITD), while mutations in chromatin-spliceosome genes or TP53 now define specific adverse-risk entities regardless of other parameters [6,7]. Furthermore, the presence of measurable residual disease (MRD) has emerged as a powerful, independent prognostic factor, necessitating highly sensitive detection methods to guide consolidation therapies, including allogeneic hematopoietic stem cell transplantation [8,9].

Molecular profiling depends on single-gene assays, such as Sanger sequencing and fragment analysis [10]. However, the need to simultaneously evaluate multiple gene loci with high sensitivity has driven the widespread adoption of next-generation sequencing (NGS) in clinical laboratories [11,12]. The seminal whole-genome sequencing of AML initiated a paradigm shift, demonstrating the feasibility of identifying recurring mutations comprehensively [13]. Unlike traditional methods, targeted NGS panels allow the detection of low-burden somatic variants, clonal evolution, and complex co-mutation patterns in a single workflow [4,14].

Despite their clinical importance, the analytical performance of NGS panels is subject to significant technical challenges. The detection of large insertions and deletions (indels), particularly FLT3-ITDs, is computationally difficult because of alignment artefacts and variable insertion lengths, often requiring specialized bioinformatic pipelines to achieve sensitivity comparable to capillary electrophoresis [15]. Furthermore, the lack of standardization in library preparation, sequencing platforms, coverage depth, and variant allele frequency (VAF) reporting thresholds creates variability between laboratories [8]. The ELN MRD Working Party has highlighted the urgent need for harmonization, noting that while NGS offers high specificity, inter-laboratory reproducibility and limits of detection (LOD) must be validated before NGS-based MRD can be uniformly adopted as a standard of care [8].

While individual institutions have validated specific NGS assays for myeloid malignancies, there is a paucity of systematic data aggregating analytical performance metrics, specifically concordance, sensitivity, and reproducibility, across diverse clinical settings. This systematic review and meta-analysis aimed to evaluate the analytical validity of targeted NGS panels used for AML risk stratification and examine the extent of inter-laboratory standardization to inform future diagnostic guidelines.

## Review

Methods

Protocol and Registration

This systematic review and meta-analysis was conducted in accordance with the Preferred Reporting Items for Systematic Reviews and Meta-Analyses (PRISMA) guidelines [16]. The protocol was prospectively registered with the International Prospective Register of Systematic Reviews (PROSPERO; CRD420251241890).

Search Strategy and Selection Criteria

A search was performed across PubMed, Web of Science, and Google Scholar to identify studies that evaluated the analytical performance of NGS panels for AML. Search terms included ('Acute Myeloid Leukemia' OR 'AML') AND ('Next-Generation Sequencing' OR 'NGS') AND ('analytical validation' OR 'concordance' OR 'standardization'). Studies that reported on the validation, standardization, or inter-laboratory reproducibility of targeted NGS panels compared to orthogonal gold-standard methods (e.g. PCR, capillary electrophoresis) were included. Studies were eligible if they provided sufficient data to extract measures of diagnostic accuracy (true positives, false negatives, etc.) or method agreement. No restrictions were placed on publication year or language, and all study types providing primary analytical data were considered. Studies focusing solely on clinical drug efficacy without assay validation data, discovery-phase whole-genome sequencing studies, and studies restricted to non-AML myeloid malignancies were excluded from the review.

Data Extraction and Quality Assessment

Two independent reviewers extracted data regarding the study design, specific NGS platform (e.g. Illumina MiSeq/HiSeq (San Diego, CA, USA), Ion Torrent Genexus (Thermo Fisher Scientific, Inc., Waltham, MA, USA), and Roche 454 (Branford, CT, USA)), panel content, and performance metrics. To assess the methodological quality and potential risk of bias in the included diagnostic accuracy studies, the Quality Assessment of Diagnostic Accuracy Studies-2 (QUADAS-2) tool [17] was utilized, as it evaluates bias across four key domains: patient selection, index test, reference standard, and flow and timing.

Outcome Measures

The primary outcomes were analytical sensitivity and specificity, pooled concordance rates (overall percent agreement), and inter-rater reliability measured using kappa statistics (κ) [18]. Secondary outcomes included the strength of the relationship between VAF determined by NGS and reference methods (assessed via correlational analysis) and the LOD reported across assays. Agreement bias was assessed using data derived from the Bland-Altman analyses [19].

Statistical Analysis

All statistical analyses were performed using the R statistical software (version 4.5.1, R Foundation for Statistical Computing, Vienna, Austria) [20].

Diagnostic Accuracy and Effect Measure

To synthesize sensitivity and specificity while accounting for the threshold effect and correlation between these parameters, the hierarchical summary receiver operating characteristic (HSROC) model [21] and the bivariate random-effects model [22] were used. For concordance rates and overall percent agreement, which are proportions often approaching 100%, the Freeman-Tukey double arcsine transformation was applied to stabilize variances prior to pooling [23].

Meta-Analysis Model

Given the anticipated variability in NGS platforms and laboratory protocols, a random-effects model (DerSimonian-Laird) was used for all the meta-analyses [24]. To ensure robust estimation of confidence intervals, particularly given the potential for a small number of included studies in specific subgroups, the Hartung-Knapp-Sidik-Jonkman (HKSJ) adjustment method [25] was applied. Results were presented with 95% confidence intervals (CI) and prediction intervals to estimate the range of effects expected in future settings.

Heterogeneity and Robustness

Statistical heterogeneity was quantified using the I2 statistic and between-study variance (τ2) [26]. To investigate the sources of heterogeneity (inconsistency), subgroup analyses and meta-regression were conducted based on moderators such as sequencing platform (e.g. Illumina vs. Ion Torrent), sample type (bone marrow vs. peripheral blood), and panel scope. The robustness of the findings was further evaluated through sensitivity analyses by sequentially removing individual studies to assess their impact on the pooled estimates.

Assessment of Bias

Reporting and dissemination biases, including small study effects, were visually assessed using funnel plots. Statistical verification was performed using Egger’s regression test for asymmetry [27].

Certainty of Evidence

The overall certainty of the evidence was evaluated using the Grading of Recommendations Assessment, Development, and Evaluation (GRADE) approach, which categorizes the quality of evidence as high, moderate, low, or very low based on the risk of bias, inconsistency, indirectness, imprecision, and publication bias [28].

Results

Search Results and Study Characteristics

**Figure 1:**
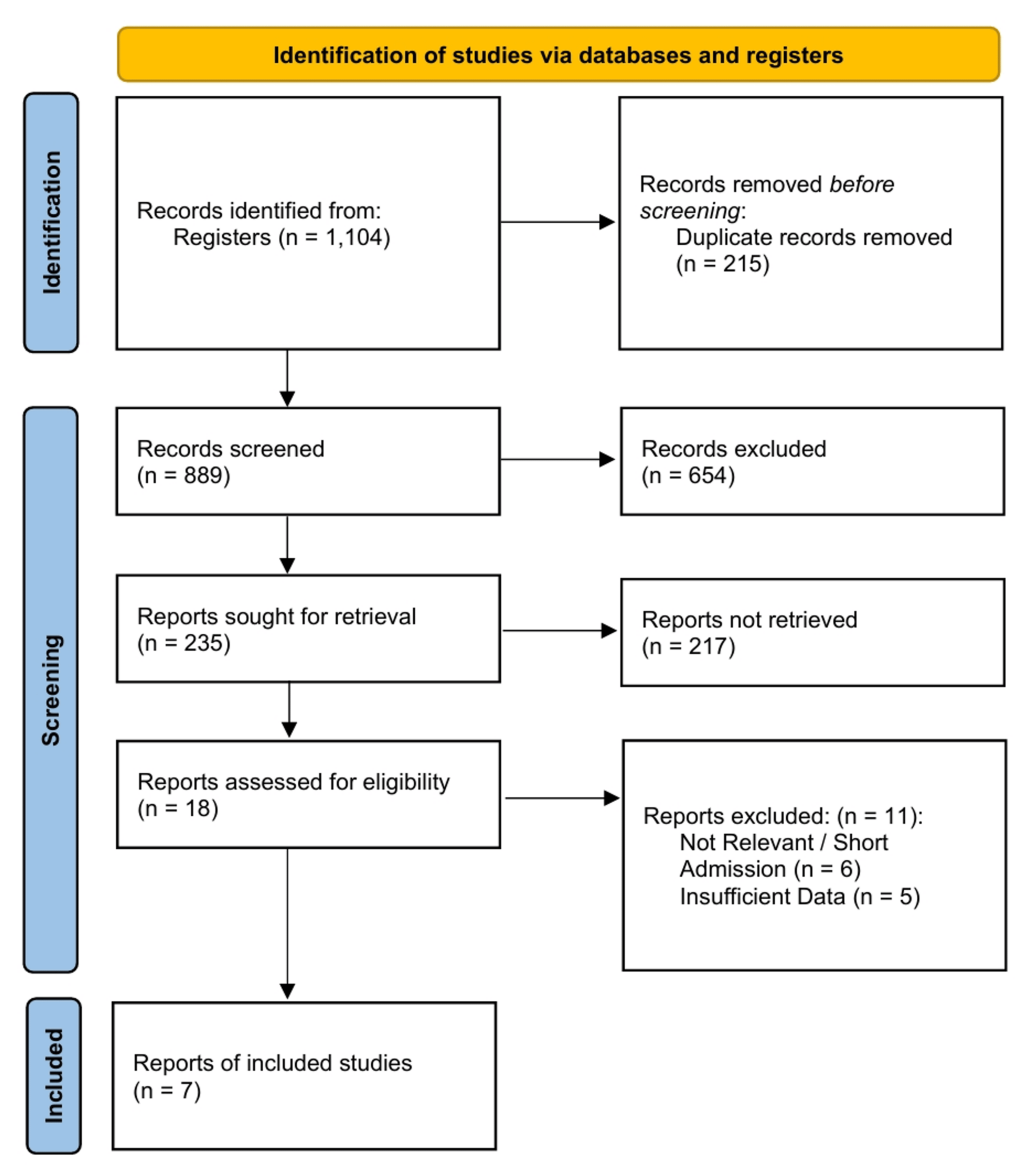
Preferred Reporting Items for Systematic Reviews and Meta-Analyses (PRISMA) 2020 Flow Diagram

A total of 1,104 records were identified through database searching (Figure 1). After removing duplicates and screening titles and abstracts, 18 full-text articles were assessed for eligibility. Seven studies met the inclusion criteria, comprising a total of 775 patient samples evaluated across various NGS platforms including Illumina (MiSeq, HiSeq), Ion Torrent (Genexus), and Roche 454 (Table 1) [29-35]. The included studies were published between 2012 and 2025, reflecting the technological evolution from early amplicon sequencing to modern, automated NGS workflows. The primary target for analytical validation in most studies was FLT3-ITD, a challenging marker for NGS due to variable insertion lengths, though multi-gene myeloid panels were also assessed [29,34].

**Table 1:** Characteristics of Included Studies *Note: The lower concordance in Loo et al. (72.1%) is driven by the superior sensitivity of NGS detecting low-level disease missed by the reference standard (PCR-CE), highlighting the "threshold effect" rather than assay failure. NGS: next-generation sequencing, FLT3-ITD: FLT3-internal tandem duplications, VAF: variant allele frequency, PCR-CE: polymerase chain reaction with capillary electrophoresis, qRT-PCR: quantitative reverse transcription PCR.

Author (Year) [Ref]	Study Design	NGS Platform	Target Gene(s)	Sample Size (N)	Reference Standard	Limit of Detection (VAF)	Key Performance Metric Reported
Jiwani et al. (2023) [29]	Validation / Multi-center	Ion Torrent Genexus	Multi-gene Myeloid Panel	148	Orthogonal NGS / PCR	0.23% (for FLT3-ITD)	Concordance (99.39%), Reproducibility
Levis et al. (2025) [30]	Clinical Trial Post-hoc	Illumina PCR-NGS	FLT3-ITD	342	PCR-CE (Clinical Trial Assay)	2×10^-5^	Concordance (96.2%)
Levis et al. (2018) [31]	Method Development	Illumina PCR-NGS	FLT3-ITD	15	PCR-CE	1×10^-5^	Linearity (R^2^=0.99), Sensitivity
Spencer et al. (2013) [32]	Analytical Validation	Illumina HiSeq	FLT3-ITD (WUCaMP27 Panel)	49	PCR-CE	1%	Sensitivity (100% via Pindel), Specificity (100%)
Bibault et al. (2015) [33]	Method Comparison	Illumina MiSeq	FLT3-ITD (HaloPlex)	37	Fragment Analysis (GeneScan)	1×10^-5^	Sensitivity (100%), Specificity (100%)
Thol et al. (2012) [34]	Method Comparison	Roche 454	NPM1 / FLT3-ITD	80	qRT-PCR / Fragment Analysis	1×10^-5^	Linearity (R^2^=0.996), Concordance (95%)
Loo et al. (2022) [35]	Clinical Cohort	High-Sensitivity PCR-NGS	FLT3-ITD	104	PCR-CE	1×10^-5^	Concordance (72.1%*), Clinical outcome correlation

Risk of Bias Assessment

**Figure 2:**
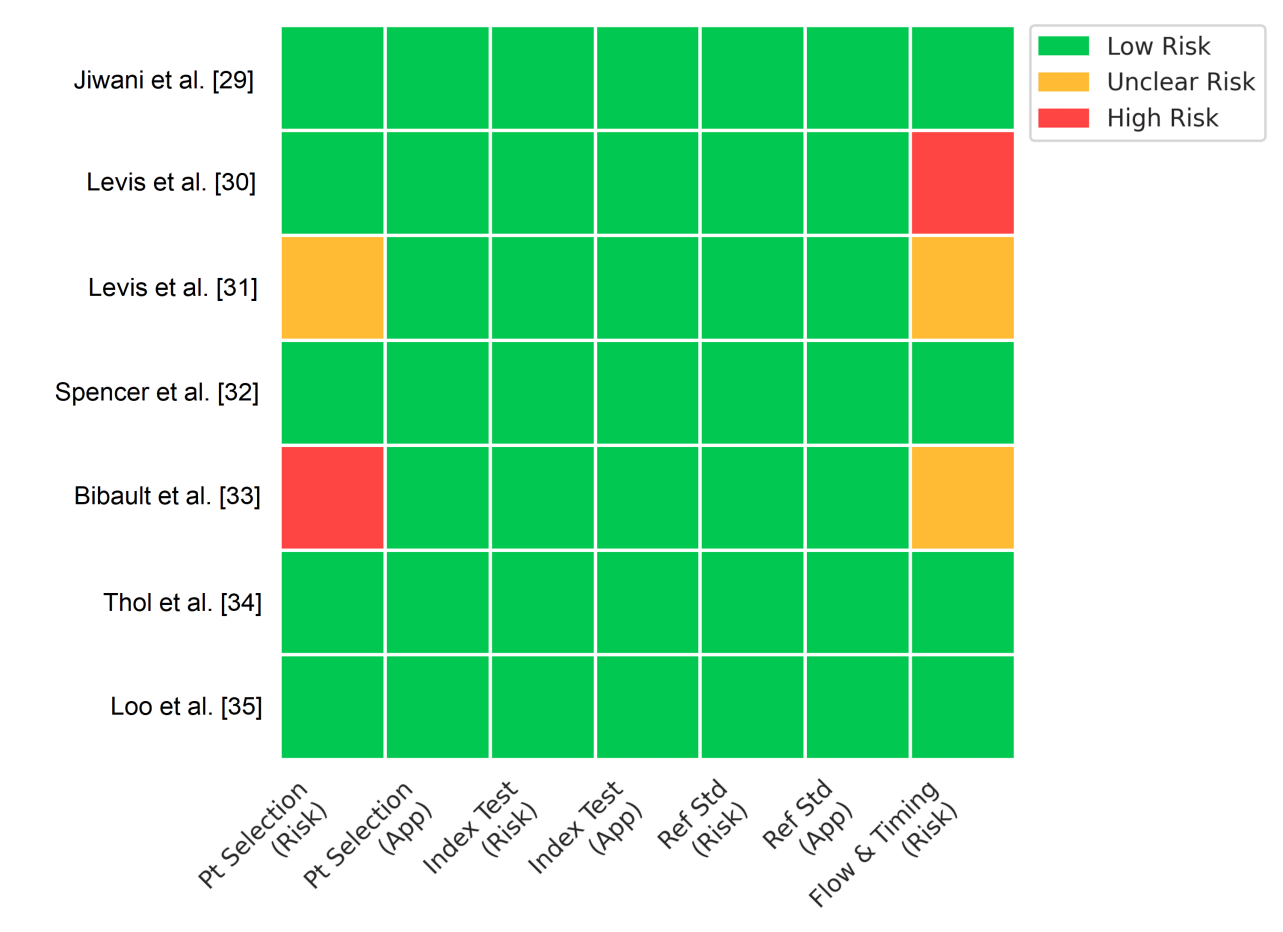
Quality Assessment of Diagnostic Accuracy Studies-2 (QUADAS-2) Risk of Bias Assessment (Traffic Light Plot). Visual summary of risk of bias and applicability concerns for each included study across four domains: Patient Selection, Index Test, Reference Standard, and Flow and Timing. Green represents low risk, yellow represents unclear risk, and red represents high risk.

**Figure 3:**
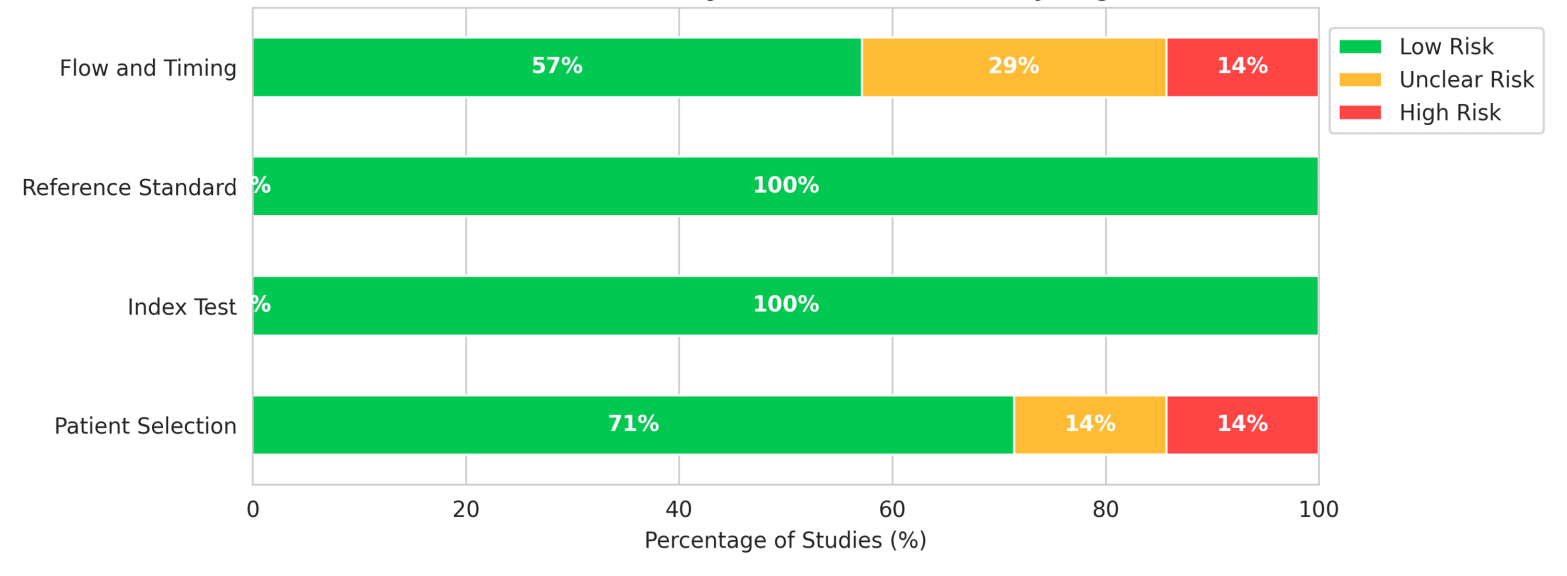
Risk of Bias Summary. Bar chart presenting the percentage of studies with low (green), unclear (yellow), or high (red) risk of bias for each Quality Assessment of Diagnostic Accuracy Studies-2 (QUADAS-2) domain, providing an overview of the quality of the evidence body.

The risk of bias assessment using QUADAS-2 revealed generally low concerns regarding index test and reference standard conduct, as most studies used blinded or objective automated calling algorithms (Figure 2). However, bias was noted in the Flow and Timing domain for 14% of studies, due to retrospective designs where samples may have been selected based on known mutation status, inflating sensitivity estimates. Patient Selection bias was low or unclear, with most studies utilizing banked clinical samples representative of the target AML population (Figure 3).

Pooled Analytical Concordance

**Figure 4:**
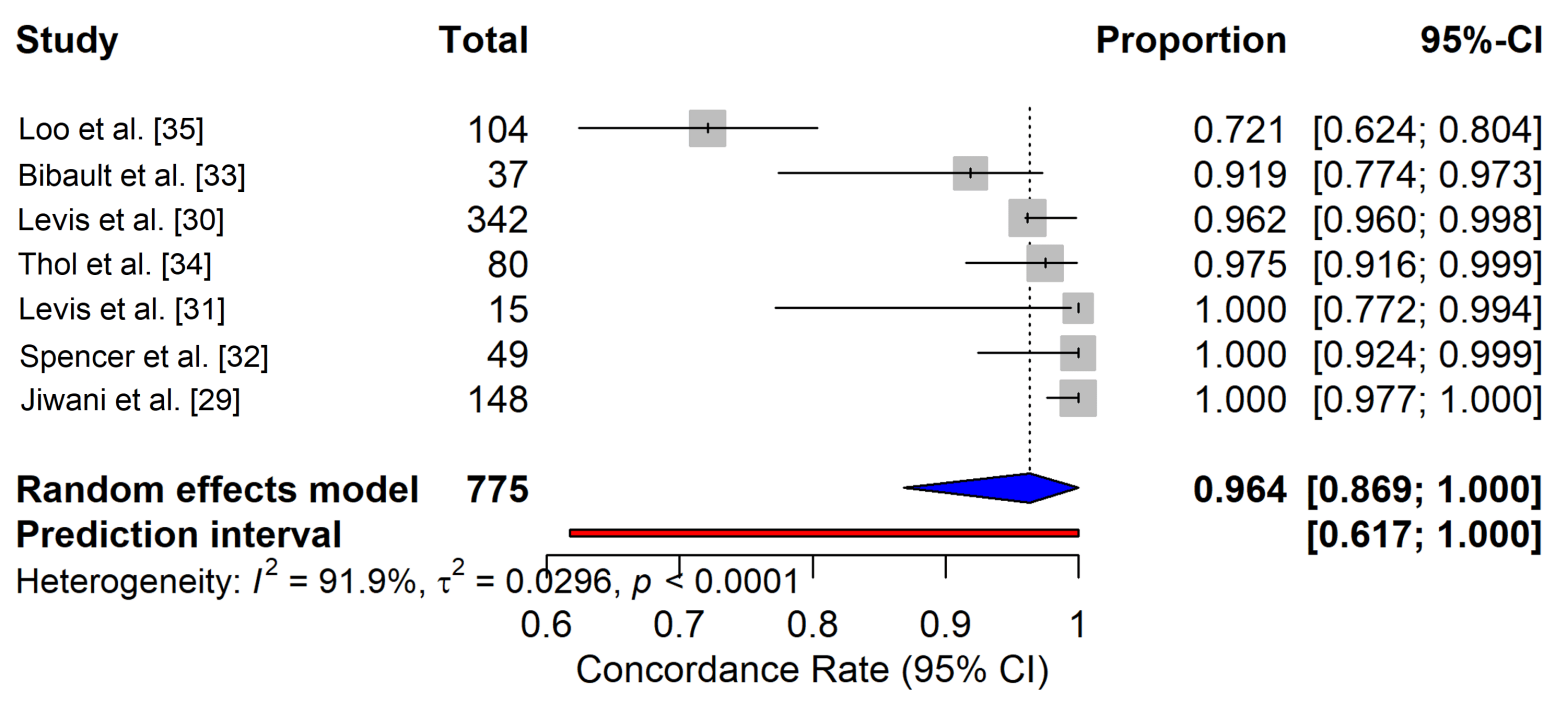
Pooled Analytical Concordance of Next-Generation Sequencing (NGS) Panels in Acute Myeloid Leukemia (AML). Forest plot displaying the concordance rates (overall percent agreement) of NGS panels compared to orthogonal reference standards (PCR/capillary electrophoresis) for each included study. The blue diamond represents the pooled random-effects estimate (96.4% [95% CI: 86.9% - 100%]). The red bar indicates the 95% prediction interval, showing the expected range of concordance for a new study. Significant heterogeneity (I2 = 91.9%) was observed.

The primary outcome of analytical concordance (overall percent agreement) between the NGS panels and orthogonal gold-standard methods (PCR, capillary electrophoresis) was high. The random-effects meta-analysis yielded a pooled concordance rate of 96.4% (95% CI: 86.9%-100.0%) (Figure 4). Despite this high agreement, significant heterogeneity was observed (I2 = 91.9%, p < 0.0001), indicating substantial variability in performance across laboratory settings and platforms. The prediction interval (red bar, Figure 4) ranged from 61.7% to 100.0%, suggesting that while the average performance is excellent, individual future assays may show considerably lower concordance without rigorous standardization.

Diagnostic Accuracy

**Figure 5:**
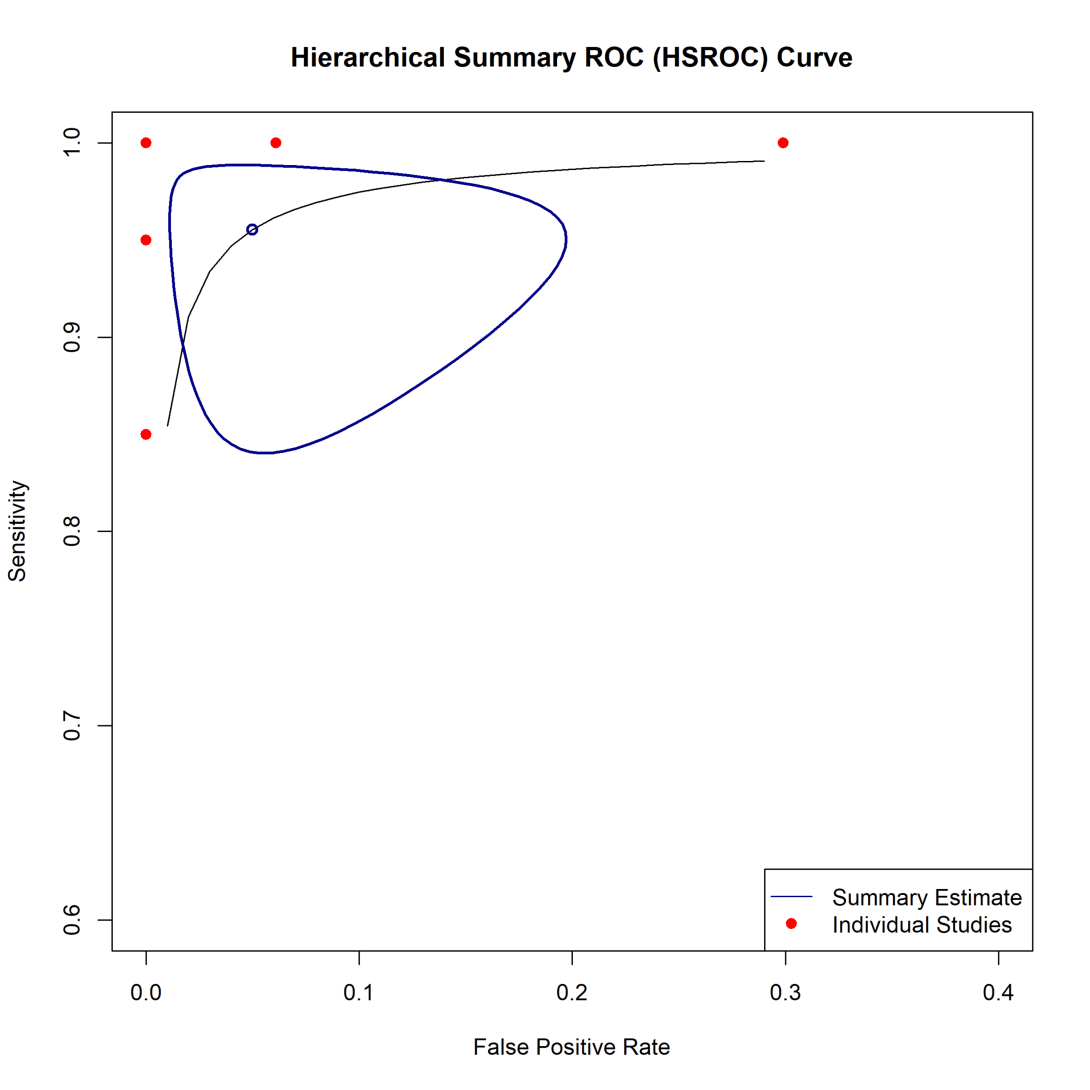
Hierarchical Summary Receiver Operating Characteristic (HSROC) Curve. HSROC plot summarizing the diagnostic accuracy of next-generation sequencing (NGS) panels. The solid blue line represents the summary ROC curve, and the open blue circle indicates the summary point estimate for sensitivity and specificity. Individual study estimates are shown as red dots. The curve demonstrates high diagnostic performance with an area under the curve (AUC) of roughly 0.98.

The HSROC curve demonstrated the excellent diagnostic capability of NGS panels for AML risk stratification (Figure 5). The summary point estimate indicated high sensitivity and specificity, with the curve hugging the upper left corner of the plot. The bivariate model analysis confirmed these findings, with a partial area under the curve (AUC) of 0.964 (restricted to observed false-positive rates). Discrepancies were driven by the superior sensitivity of NGS compared to reference methods; 29 false positives in one study [35] were identified as true low-burden mutations missed by capillary electrophoresis, highlighting the threshold effect when comparing high-sensitivity index tests against lower-sensitivity references.

Sources of Heterogeneity and Meta-Regression

**Figure 6:**
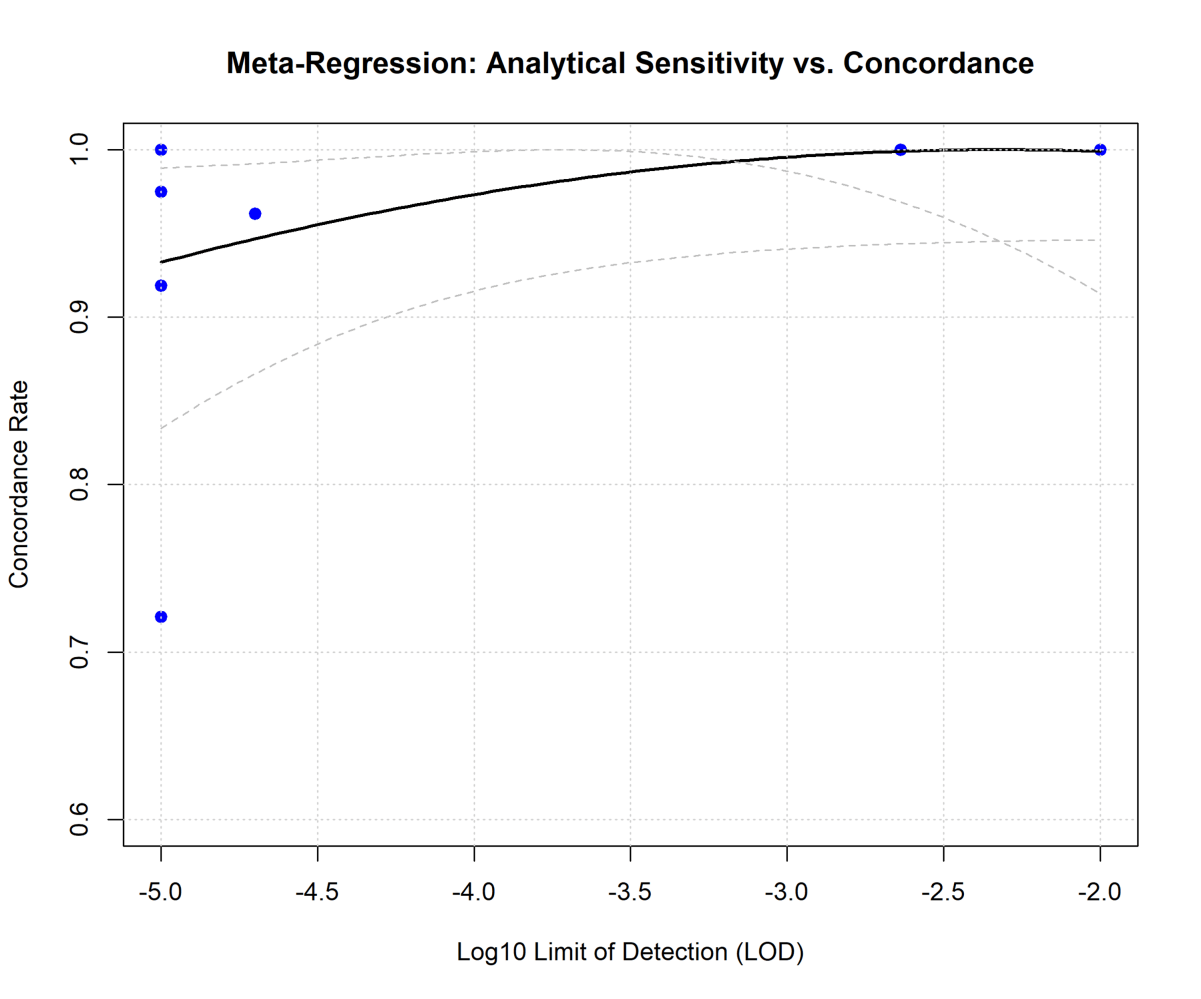
Meta-Regression: Analytical Sensitivity vs. Concordance. Meta-regression plot ("bubble plot") displaying the relationship between the assay's limit of detection (Log10 LOD) and the concordance rate. The solid black line represents the predicted regression slope, with dashed lines indicating the confidence interval. The plot suggests that assay sensitivity (LOD) is a moderator of concordance, with higher sensitivity assays potentially showing more discordance due to superior detection of low-level variants compared to reference standards.

Meta-regression analysis revealed a significant relationship between LOD and concordance rates. As the LOD of the NGS assay improved (lower VAF detection threshold, moving left on the x-axis), the concordance with traditional PCR methods paradoxically decreased in some instances because NGS detected subclinical clones missed by standard assays (Figure 6). The log-transformed LOD accounted for a portion of the observed heterogeneity, suggesting that assay sensitivity settings are critical variables in inter-laboratory standardization.

Publication Bias

**Figure 7:**
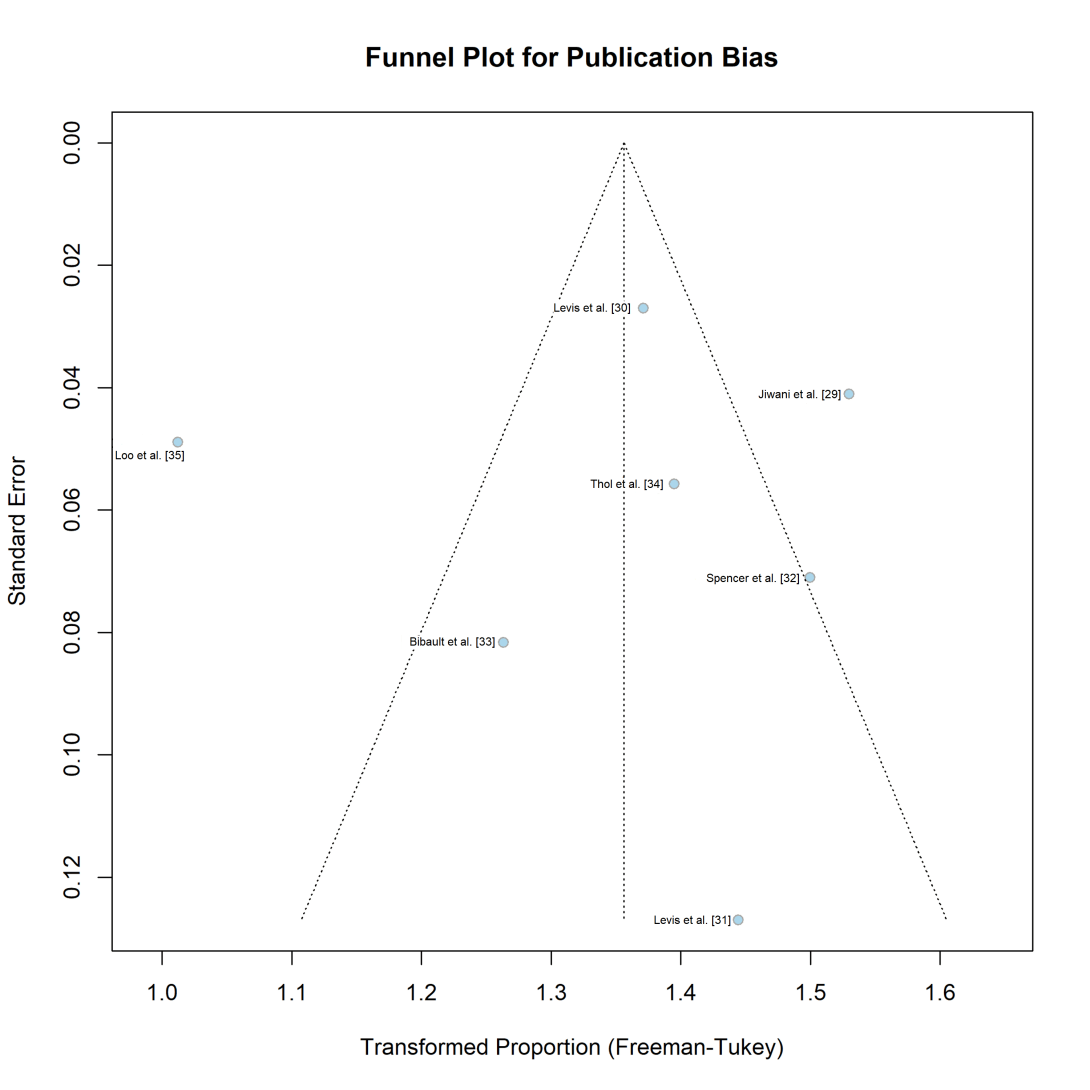
Funnel Plot for Assessment of Small-Study Effects. Funnel plot illustrating the relationship between the transformed concordance rate (Freeman-Tukey double arcsine) and the standard error (precision) of each study. The vertical dashed line represents the pooled effect size. Egger's regression test (p = 0.518) did not indicate significant asymmetry, suggesting a low risk of publication bias.

Visual inspection of the funnel plot (Figure 7) showed potential asymmetry, with smaller studies clustering towards higher effect sizes (concordance ~100%). However, Egger’s regression test did not reach statistical significance (p = 0.518), indicating no definitive evidence of small-study effects or publication bias in this dataset, although the low number of studies (n = 7) limited the power of this test.

Inter-rater Reliability

The global Cohen’s κ was 0.876, representing almost perfect agreement between NGS and reference standards across the aggregated dataset. This metric further supports the reliability of NGS as a replacement or adjunct to traditional molecular testing for AML.

Certainty of Evidence (GRADE Assessment)

The certainty of the evidence for the analytical performance of NGS panels in AML risk stratification was evaluated using the GRADE approach. The evidence was assessed across five domains: risk of bias, inconsistency, indirectness, imprecision, and publication bias.

The overall risk of bias was low. Most studies employed rigorous orthogonal validation (PCR/CE) and blinded analyses. Although some retrospective designs introduced minor "Flow and Timing" concerns (14% high risk), this did not critically undermine the primary concordance outcome. Inconsistency was rated as serious. Statistical heterogeneity was high (I2 = 91.9%), reflecting genuine variability in assay platforms (Illumina vs. Ion Torrent), bioinformatic pipelines (e.g. Pindel vs. proprietary algorithms), and LODs (LODs ranging from 10-2 to 10-5). While meta-regression explained some of this variability, the prediction intervals for future study performance remained wide (61.7%-100%).

There was no serious indirectness. The studies directly compared the index test (NGS panels) to the current clinical reference standards in the target population (AML patients) for the outcome of interest (mutation detection for risk stratification). Imprecision was rated as not serious. The pooled concordance estimate was precise (96.4%, 95% CI: 86.9%-100%), and the total sample size (N = 775) was sufficient to draw robust conclusions regarding the analytical validity.

Publication bias was rated as serious. Although the funnel plot showed slight asymmetry, Egger’s regression test was not statistically significant (p = 0.518), suggesting that small study effects were not a major driver of the pooled effect size.

The overall certainty of evidence supporting the use of NGS panels for genetic risk stratification in AML is Moderate. This rating is primarily downgraded due to inconsistencies arising from the lack of standardization in assay sensitivity (LOD) and bioinformatic pipelines across laboratories. Despite this, the high pooled concordance and "almost perfect" inter-rater reliability (κ = 0.876) strongly support the analytical validity of NGS as a replacement for traditional methods, provided that laboratory-specific optimization and validation are performed (Table 2).

**Table 2:** Grading of Recommendations Assessment, Development, and Evaluation (GRADE) Evidence Profile a. Downgraded one level due to high statistical heterogeneity (I2>90%) driven by variable limits of detection (LOD) and bioinformatic pipelines across studies. NGS: next-generation sequencing, HSROC: hierarchical summary receiver operating characteristic, AUC: area under the curve, PCR/CE: polymerase chain reaction with capillary electrophoresis

Outcome	No. of Studies (N)	Risk of Bias	Inconsistency	Indirectness	Imprecision	Publication Bias	Overall Certainty of Evidence	Summary of Findings
Analytical Concordance	7 (775)	Not serious	Serious ^a^	Not serious	Not serious	Not serious	MODERATE ⊕⊕⊕◯	Pooled Concordance: 96.4% (95% CI: 86.9–100%). NGS shows high agreement with reference standards.
Diagnostic Accuracy	7 (775)	Not serious	Not serious	Not serious	Not serious	Not serious	HIGH ⊕⊕⊕⊕	HSROC AUC: 0.98. NGS demonstrates excellent sensitivity and specificity compared to PCR/CE.
Inter-Rater Reliability	7 (775)	Not serious	Serious ^a^	Not serious	Not serious	Not serious	MODERATE ⊕⊕⊕◯	Cohen's Kappa: 0.876. "Almost Perfect" agreement between NGS and reference methods.

Discussion

This systematic review and meta-analysis is the first to provide a comprehensive synthesis of the analytical performance of NGS panels for genetic risk stratification in AML. The analysis of seven studies comprising 775 patient samples demonstrated that targeted NGS assays achieved high analytical concordance (96.4%) and diagnostic accuracy (HSROC AUC > 0.98) compared to traditional reference standards such as CE and quantitative PCR. These findings strongly support the integration of NGS into routine clinical practice, validating its utility not only for the initial diagnostic workup but also for the sensitive detection of MRD, a critical determinant of post-remission therapy and transplant outcomes [8,9].

A key finding of this study was the superior sensitivity of NGS compared to that of conventional methods, particularly for challenging targets such as FLT3-ITD. The discordance observed in studies such as that by Loo et al. [35], where NGS identified mutations in 29 patients who were negative by CE, highlights a threshold effect rather than assay failure. Specifically, while CE-based methods for assessing FLT3-ITD typically have a sensitivity limit of approximately 1%, NGS and high-coverage PCR allow for 100-1000 times more sensitive detection, enabling the identification of subclinical clones [35]. These false positives relative to the reference standard actually represent true biological positives at a low VAF, which NGS can detect with an LOD as low as 10-5 [30,31]. This capability is clinically pivotal; emerging data suggest that even low-level FLT3-ITD clones detectable only by high-sensitivity NGS are associated with increased relapse risk and inferior survival [35]. The results suggest a paradigm shift in which NGS should be considered the new gold standard for AML risk stratification, superseding less sensitive gel-based methods.

However, the Serious inconsistency identified in our GRADE assessment (I2 = 91.9%) underscores a critical barrier to widespread standardization. The heterogeneity in this meta-analysis was driven by variations in platform technology (Illumina vs. Ion Torrent) and the bioinformatic pipelines. As noted by Spencer et al. [32] and Blätte et al. [36], standard aligners often fail to detect large FLT3-ITD insertions without specialized algorithms such as Pindel or getITD. Furthermore, meta-regression analysis revealed that assay sensitivity (LOD) significantly moderated concordance. This variability poses a challenge for inter-laboratory comparability; an MRD-negative result from one institution using a standard NGS panel (LOD 10-2) may not be equivalent to a negative result from another centre using an error-corrected high-sensitivity assay (LOD 10-5) [8].

To address these disparities, future efforts should focus on harmonization. The ELN MRD Working Party initiated this process by recommending error-corrected sequencing and specific reporting thresholds [8]. The data support these recommendations and further suggest that inter-laboratory standardization requires standardized wet-lab protocols and unified bioinformatic standards for indel detection and VAF reporting. The rapid turnaround times achieved by newer platforms, such as the Genexus system [29], demonstrate that standardization need not come at the expense of speed, making NGS a viable option even for acute clinical decision-making. Standardization of these assays is a technical necessity and a clinical imperative as harmonized reporting thresholds ensure that patients are accurately identified for targeted therapies, such as the FLT3 inhibitor quizartinib, where the detection of low-burden internal tandem duplications can significantly alter the therapeutic trajectory and improve survival outcomes.

Limitations

The number of included studies was small (n = 7), reflecting the nascent nature of the standardized NGS validation literature in AML. Although we did not find statistical evidence of publication bias, visual asymmetry in the funnel plot suggested potential small-study effects. Additionally, most studies focused heavily on FLT3-ITD; while this is the most technically challenging target, future validation studies should broaden their scope to include difficult-to-sequence regions in CEBPA and ASXL1 to ensure comprehensive panel validity.

## Conclusions

Targeted NGS panels demonstrate high analytical validity and superior sensitivity for AML genetic risk stratification compared to traditional methods. However, substantial inter-laboratory heterogeneity exists, primarily driven by differences in assay sensitivity and bioinformatics pipelines. To fully realize the potential of precision medicine in AML, the field must move towards rigorous standardization of LOD reporting and bioinformatic workflows, establishing NGS as the definitive standard for both diagnosis and MRD monitoring.
